# Return of chloroquine sensitivity to Africa? Surveillance of African *Plasmodium falciparum* chloroquine resistance through malaria imported to China

**DOI:** 10.1186/s13071-017-2298-y

**Published:** 2017-07-26

**Authors:** Feng Lu, Meihua Zhang, Richard L. Culleton, Sui Xu, Jianxia Tang, Huayun Zhou, Guoding Zhu, Yaping Gu, Chao Zhang, Yaobao Liu, Weiming Wang, Yuanyuan Cao, Julin Li, Xinlong He, Jun Cao, Qi Gao

**Affiliations:** 1 Key Laboratory of National Health and Family Planning Commission on Parasitic Disease Control and Prevention, Jiangsu Provincial Key Laboratory on Parasite and Vector Control Technology, Jiangsu Institute of Parasitic Diseases, Wuxi, 214064 Jiangsu Province, People’s Republic of China; 2 Department of Pathogen Biology and Immunology, School of Medicine, Yangzhou University, Jiangsu Key Laboratory of Experimental & Translational Non-coding RNA Research, Yangzhou, 225001 Jiangsu Province, People’s Republic of China; 3 Malaria Unit, Department of Pathology, Institute of Tropical Medicine, Nagasaki University, Sakamoto, Nagasaki, 852-8501 Japan; 4 The Third People’s Hospital of Wuxi, Wuxi, 214041 Jiangsu Province, People’s Republic of China; 5 Public Health Research Center, Jiangnan University, Wuxi, 214122 Jiangsu Province, People’s Republic of China

**Keywords:** *Plasmodium falciparum*, Chloroquine, Drug-resistance, *Pfcrt*

## Abstract

**Background:**

Chloroquine (CQ) was the cornerstone of anti-malarial treatment in Africa for almost 50 years, but has been widely withdrawn due to the emergence and spread of resistance. Recent reports have suggested that CQ-susceptibility may return following the cessation of CQ usage. Here, we monitor CQ sensitivity and determine the prevalence of genetic polymorphisms in the CQ resistance transporter gene (*pfcrt*) of *Plasmodium falciparum* isolates recently imported from Africa to China.

**Methods:**

Blood samples were collected from falciparum malaria patients returning to China from various countries in Africa. Isolates were tested for their sensitivity to CQ using the SYBR Green I test ex vivo, and for a subset of samples, in vitro following culture adaptation. Mutations at positions 72–76 and codon 220 of the *pfcrt* gene were analyzed by sequencing and confirmed by PCR-RFLP. Correlations between drug sensitivity and *pfcrt* polymorphisms were investigated.

**Results:**

Of 32 culture adapted isolates assayed, 17 (53.1%), 6 (18.8%) and 9 (28.1%) were classified as sensitive, moderately resistant, and highly resistant, respectively. In vitro CQ susceptibility was related to point mutations in the *pfcrt* gene, the results indicating a strong association between *pfcrt* genotype and drug sensitivity. A total of 292 isolates were typed at the *pfcrt* locus, and the prevalence of the wild type (CQ sensitive) haplotype CVMNK in isolates from East, South, North, West and Central Africa were 91.4%, 80.0%, 73.3%, 53.3% and 51.7%, respectively. The only mutant haplotype observed was CVIET, and this was almost always linked to an additional mutation at A220S.

**Conclusions:**

Our results suggest that a reduction in drug pressure following withdrawal of CQ as a first-line drug may lead to a resurgence in CQ sensitive parasites. The prevalence of wild-type *pfcrt* CQ sensitive parasites from East, South and North Africa was higher than from the West and Central areas, but this varied greatly between countries. Further surveillance is required to assess whether the prevalence of CQ resistant parasites will continue to decrease in the absence of widespread CQ usage.

## Background

Among the five species of *Plasmodium* that cause malaria in humans, *Plasmodium falciparum* is the most prevalent and virulent, causing high levels of mortality and morbidity worldwide, particularly in sub-Saharan Africa. At present, malaria control relies on antiparasitic drugs and anti-mosquito measures. However, the emergence, selection and spread of drug-resistant *P. falciparum* threatens malaria control and elimination efforts, and there is a growing concern that resistance against the most effective drug currently available, artemisinin and its derivatives, is emerging [1]. In order to control malaria, it is important to prolong the life span of the drugs currently used against the disease.

Chloroquine (CQ), was the most frequently used first-line therapy for uncomplicated *P. falciparum* malaria from the 1940s through to the 2000s due to its high efficacy, safety and low cost [2]. Resistance to CQ was first identified on the Thai-Cambodian border in the late 1950s, concomitantly in South America, and in Africa in the 1970s [3–5]. The spread of chloroquine resistance has led to the promotion of sulfadoxine-pyrimethmine (SP) and artemisinin-based combination therapies as first-line treatments for uncomplicated malaria [6, 7]. The associated reduction in CQ use has been linked to the return of CQ susceptibility in several malaria-endemic countries [8]. It has been argued that the cessation of the use of CQ until sensitive parasites reemerge may allow the drug to be reintroduced in these areas [9]. In order to determine whether resistant parasites are disappearing from areas in the absence of CQ use, molecular markers of resistance must be assayed from these populations.

Although the emergence of spread of CQ resistance has multi-factorial causes, it is known that mutations in the *P. falciparum* CQ resistance transporter gene (*pfcrt*), are important for the parasite’s acquisition of resistance. A single nucleotide mutation which results in change from lysine to threonine at position 76 of the PfCRT protein is known to confer CQ resistance (CQR), and is the most reliable molecular marker for its presence [10–12]. The K76T mutation acts in conjunction with other mutations [13, 14]; codons 74, 75, 220, 271, 326 and 371 have also been shown to play a role in in vitro CQ-resistance [11, 15, 16].

In China, while the number of autochthonous cases has recently declined, there has been a steady increase in the numbers of cases of imported malaria, the majority of which occur in returning laborers. This has become a major challenge for malaria elimination in China [17]. These cases are mainly acquired from African countries, and *P. falciparum* is the most common species. To inform current and future guidelines on antimalarial use in China, and also African countries, we conducted a CQ sensitivity study and a survey of *pfcrt* haplotypes in imported *P. falciparum* isolates from African countries.

## Methods

### Parasite samples and DNA extraction

This study was carried out in Jiangsu Province, China, where only imported malaria cases have been reported in recent years. Blood samples were collected from 2012 to 2014 from malaria patients with uncomplicated *P. falciparum* infections at local hospitals or centers for disease control and prevention in Jiangsu Province. Malaria infection was initially diagnosed by microscopy of Giemsa’s solution-stained thick and thin blood smears. Upon confirmation of *P. falciparum*, 2 ml of venous blood was obtained from each patient by venipuncture into a heparinized tube, blood was stored at 37 °C and transported to the laboratory for in vitro adaptation within 24 h of collection. Blood filter papers from each patient were also prepared, and stored individually after being air dried for later analyses. Genomic DNA was extracted from venous blood samples and filter papers using QIAamp DNA blood kit (Qiagen, Valencia, CA), as per the manufacturer’s protocol. Malaria parasite species identification was carried out by nested PCR as previously described [18]. Only samples confirmed as mono-infections of *P. falciparum* were included in this study.

### In vitro adaptation of parasites and chloroquine susceptibility assay

Parasites were adapted to continuous culture immediately upon receipt of blood from patients. Blood samples were treated as described previously [19], except that a non-woven fabric (NWF) filter was used to remove leukocytes [20]. Samples were suspended at a 2% hematocrit in complete medium with HEPES (5.94 g/l), hypoxanthine (50 mg/l), Albumax I (5 g/l), RPMI 1640 (10.4 g/l), gentamicin (5 mg/l), and NaHCO_3_ (2.1 g/l). The mixture was incubated in a tri-gas incubator (containing 5% CO_2_, 5% O_2_, and 90% N_2_) at 37 °C. Routine culture of the parasites was maintained with type O^+^ RBCs.

Only samples from patients with parasitaemias ≥ 1%, who had not received antimalarial treatment in the preceding three weeks, and when blood samples were transported to the lab within four hours were chosen for ex vivo drug assays. All samples were selected for drug assay after in vitro adaptation, which involved four to five weeks of continuous culture, on average.

The CQ susceptibility of *P. falciparum* isolates was measured using the World Health Organization protocol [21], with some minor modifications. Chloroquine diphosphate (molecular weight [MW], 515.9; Sigma, St. Louis, MO, USA) was prepared by dilution with distilled water, and further diluted with 70% ethanol to achieve a concentration series of 12.5–800 nM in a 96-well culture plate as described previously [22]. This pre-dosed plate was dried, sealed, and stored at 4 °C for up to three months. One hundred microliters of synchronized ring-stage parasite suspension were dispensed into triplicate wells of a pre-dosed CQ plate to obtain a 2% hematocrit and 0.5–1.0% parasitemia. The plates were shaken for 5 min and incubated in a candle jar at 37 °C for 72 h. Chloroquine effectiveness was measured by the SYBR green I-based fluorescence assay [23]. The laboratory clone 3D7 and K1 were included throughout the study as controls.

### Analysis of genetic polymorphisms in the *pfcrt* gene

Polymorphisms in codons 72 to 76 and codon 220 of the *pfcrt* gene were determined using PCR and sequencing as described previously with minor adjustment [24]. The two fragments, which span codons 44–177 and 181–222 of *pfcrt*, were amplified by nested PCR assays. Amplified DNA was purified using NucleoSpin® Extract II Kits (Macherey-Nagel, Düren, Germany) according to the manual, and sequenced using an ABI PRISM®310 genetic analyzer (Applied Biosystems, Foster City, CA, USA). Sequences were aligned and analyzed using Lasergene software (DNASTAR, Madison, WI, USA). Prior to sequencing, PCR products were cloned into the pGEM-T easy vector (Promega, Madison, USA), and plasmids prepared using the Wizard Plus SV Minipreps DNA purification system (Promega). In addition, genotyping of *pfcrt* K76T by PCR-RFLP was performed for a subset of samples as described by Schneider et al. [25].

### Data analysis

Sequences of *pfcrt* were aligned and analyzed using Lasergene software. A geographical map of *pfcrt* haplotypes was produced using SmartDraw software. The geometric mean of the 50% inhibitory concentration (IC_50_), and the 95% confidence interval (CI) were determined using GraphPad Prism 5.0 for Windows. All other statistical analyses were performed using SPSS 16.0 for Windows. Differences in IC_50_ between K76 and 76T were tested using Wilcoxon rank sum test for two independent samples. Differences in IC_50_ between in vitro testing and the ex vivo test were tested using Wilcoxon signed rank test for paired samples. Mutation rates in different years were compared by Chi-square or Fisher’s exact tests. All tests were two-sided, *P*-values less than 0.05 were considered significant.

## Results

### Parasite samples collection

**Fig. 1:**
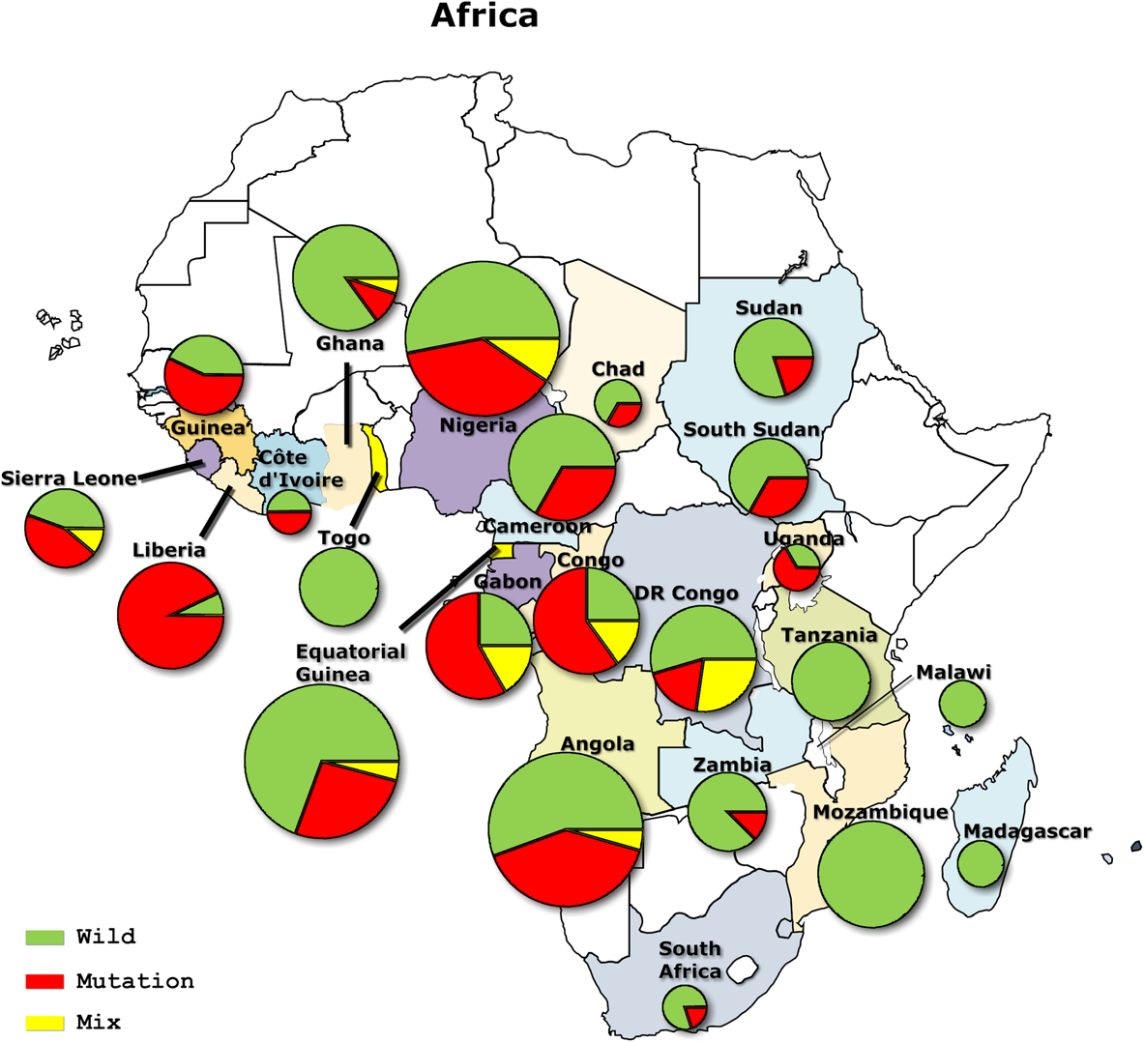
Geographical distribution of *pfcrt* haplotypes in imported *P. falciparum* isolates based on amino acid site 76. Circle sizes represent the number of samples from each site. Pie charts presenting the frequencies of the *pfcrt* haplotypes found in the imported *P. falciparum* isolates. Wild type = K76; mutant type = 76T; mixed type = K76 + T76

From 2011 to 2014, more than 1000 imported malaria cases were reported in Jiangsu Province, with *Plasmodium* species identification by nested PCR based on the 18S rRNA gene revealing that 80% were single species *P. falciparum* infections. Two hundred and ninety-two *P. falciparum* isolates, of which 29, 87, 113 and 63 were collected in 2011, 2012, 2013 and 2014, respectively, were selected for analysis of *pfcrt* polymorphism, based on the geographical area in which they were contracted. Blood samples were collected from migrant workers returning from 23 African countries (Table 1, Fig. 1). Among them were 35 isolates from six East African countries, 92 isolates from seven West African countries, five isolates from one country in South Africa, 15 isolates from two North African countries and 145 isolates from eight countries in central Africa. In addition, ten isolates were tested for ex vivo CQ sensitivity, 22 parasite isolates, including the latter ten, were culture-adapted and assayed for in vitro susceptibility to CQ using the SYBR green I method. The culture-adapted isolates were from Equatorial Guinea (*n* = 13 samples), Angola (*n* = 15), Nigeria (*n* = 1), Cameroon (*n* = 1), Sierra Leone (*n* = 1), and Gabon (*n* = 1).

**Table 1:** Distribution of *pfcrt* haplotypes in *P. falciparum* cases from different geographical regions. Total, number of samples successfully analyzed for *pfcrt* 72–76 and 220 allelic types, respective percent values in parentheses. Mutated amino acid is underlined

Area	Total	Amino acid locus					
		72–76			220		
		CVMNK	CVIET	CVMNK + CVIET	A	S	A + S
East Africa							
Madagascar	4 (1.4)	4 (100)	0 (0)	0 (0)	4 (100)	0 (0)	0 (0)
Malawi	2 (0.7)	2 (100)	0 (0)	0 (0)	2 (100)	0 (0)	0 (0)
Mozambique	12 (4.1)	12 (100)	0 (0)	0 (0)	12 (100)	0 (0)	0 (0)
Tanzania	6 (2.0)	6 (100)	0 (0)	0 (0)	6 (100)	0 (0)	0 (0)
Uganda	3 (1.0)	1 (33.3)	2 (66.7)	0 (0)	1 (33.3)	2 (66.7)	0 (0)
Zambia	8 (2.7)	7 (87.5)	1 (12.5)	0 (0)	7 (87.5)	1 (12.5)	0 (0)
West Africa							
Côte d’Ivoire	4 (1.4)	2 (50.0)	2 (50.0)	0 (0)	2 (50.0)	1 (25.0)	1 (25.0)
Ghana	20 (6.8)	17 (85.0)	2 (10.0)	1 (5.0)	17 (85.0)	2 (10.0)	1 (5.0)
Guinea	7 (2.4)	3 (42.9)	4 (57.1)	0 (0)	3 (42.9)	4 (57.1)	0 (0)
Liberia	14 (4.8)	1 (7.1)	13 (92.9)	0 (0)	1 (7.1)	12 (85.7)	1 (7.1)
Nigeria	32 (10.9)	17 (53.1)	12 (37.5)	3 (9.4)	17 (53.1)	11 (34.4)	4 (12.5)
Sierra Leone	9 (3.1)	3 (33.3)	4 (44.4)	2 (22.2)	3 (33.3)	4 (44.4)	2 (22.2)
Togo	6 (2.0)	6 (100)	0 (0)	0 (0)	6 (100)	0 (0)	0 (0)
South Africa							
South Africa	5 (1.7)	4 (80.0)	1 (20.0)	0 (0)	5 (100)	0 (0)	0 (0)
North Africa							
South Sudan	6 (2.0)	4 (66.7)	2 (33.3)	0 (0)	4 (66.7)	2 (33.3)	0 (0)
Sudan	9 (3.1)	7 (77.8)	2 (22.2)	0 (0)	7 (77.8)	2 (22.2)	0 (0)
Central Africa							
Cameroon	11 (3.7)	7 (63.6)	3 (27.3)	1 (9.1)	6 (54.5)	3 (27.3)	2 (18.2)
Angola	42 (14.3)	22 (52.4)	17 (40.5)	3 (7.1)	21 (50.0)	17 (40.5)	4 (9.5)
Chad	3 (1.0)	2 (66.7)	1 (33.3)	0 (0)	2 (66.7)	0 (0)	1 (33.3)
Congo	20 (6.6)	4 (20.0)	12 (60.0)	4 (20.0)	4 (20.0)	12 (60.0)	4 (20.0)
DR Congo	11 (3.7)	6 (54.6)	2 (18.2)	3 (27.3)	5 (45.5)	5 (27.3)	3 (27.3)
Equatorial Guinea	47 (16.0)	32 (68.1)	13 (27.7)	2 (4.3)	32 (68.1)	10 (21.3)	5 (10.6)
Gabon	11 (3.7)	2 (18.2)	7 (63.6)	2 (18.2)	2 (18.2)	7 (63.6)	2 (18.2)
Total	292 (100)	171 (58.6)	100 (34.2)	21 (7.2)	169 (57.9)	93 (31.8)	30 (10.3)

### Isolate sensitivity to CQ

Thirty-two culture-adapted isolates with single species *P. falciparum* infection were collected in 2012 (*n* = 5), 2013 (*n* = 21) and 2014 (*n* = 6). In vitro susceptibilities to CQ were assayed using the SYBR green I method. Based on the criteria described earlier, CQ susceptibility was categorized into three levels: sensitive (S: IC_50_ < 25 nM); moderately resistant (MR: 25 nM ≥ IC_50_ < 100 nM); and highly resistant (HR: IC_50_ ≥ 100 nM) [26, 27]. The median IC_50_ for CQ was 23.6 nM, ranging from 1.0 to 210.5 nM. Despite this wide variation in sensitivity, of the 32 isolates assayed, 17 (53.1%), six (18.8%) and nine (28.1%) isolates were classified as S, MR, and HR, respectively. Of the nine isolates with HR, four were from Angola, three from Equatorial Guinea, one from Gabon and one from Cameroon. In addition, ten samples were selected for ex vivo testing. Compared to the in vitro test, there was no significant differences in mean IC_50_s between them (*Z* = -0.357, *P* = 0.720); nine of these were consistent with the in vitro testing, however, the IC_50_ of one isolate was 20.4 nM by the ex vivo test, and 133.0 nM by the in vitro test (Table 2). Compared to the values of the laboratory clone 3D7 and Dd2, the IC_50_ of six isolates were lower than 3D7, and two isolates were higher than Dd2.

**Table 2:** Ex vivo and in vitro susceptibility of *Plasmodium falciparum* isolates to chloroquine

Isolate	IC_50_ (nM)	
	Ex vivo	In vitro
1	113	151.7
2	15.9	9.7
3	11.3	11.9
4	4.8	11.9
5	10.5	25
6	32.3	31.3
7	60.3	43.3
8	57.6	56.8
9	20.4	133
10	23.8	6.1

*Note*: Total ten *P. falciparum* isolates were tested for their sensitivity to chloroquine with paired fresh and culture-adapted samples, and the correlations and differences between IC_50_ values were tested, with Spearman’s correlation coefficient of 0.679 (*P* = 0.31), and there was no significant difference in mean IC_50_s between them (*Z* = -0.357, *P* = 0.720)

### Polymorphisms in the *pfcrt* gene

Polymorphisms in the *pfcrt* gene of parasites collected from all enrolled patients were analyzed successfully, with our assay covering codons 72–76 and 220 (Table 1). When mixed infections were excluded, mutations at codons 72–76 were found to be consistent with that at codon 220. The wild type haplotype C_72_V_73_M_74_N_75_K_76_ (58.6%) and A_220_ (57.9%) was the most common, followed by the mutant type C_72_V_73_I_74_E_75_T_76_ (34.2%) and S_220_ (31.8%), and others were mixed haplotypes, such as C_72_V_73_M_74_N_75_K_76_+ C_72_V_73_I_74_E_75_T_76_ (7.2%) and/or A_220_ + S_220_ (10.3%). The CVMNK haplotype was predominant in isolates from East, South and North Africa (91.4, 80.0 and 73.3%, respectively); however, its prevalence was markedly lower in West and Central Africa (53.3 and 51.7%, respectively). A higher number of mixed genotypes were found in isolates from West and Central Africa compared to elsewhere on the continent. In addition, culture-adapted parasite isolates were subject to additional sequencing of the *pfcrt* locus, the results being consistent with the original, except in the case of one isolate initially typed as a “mixed genotype”, which was found to be a pure mutant type following continuous culture adaptation. The results of the PCR-RFLP assay were entirely consistent with DNA sequencing.

### Correlation between polymorphisms in the *pfcrt* gene and in vitro sensitivity of parasite isolates to CQ

**Fig. 2:**
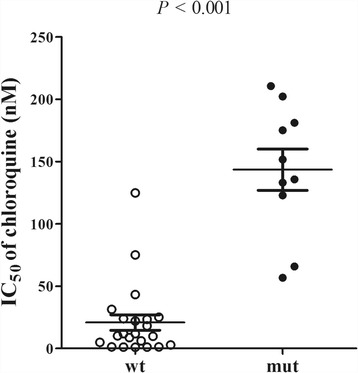
Dot plots of in vitro responses of parasite isolates to chloroquine and correlation with *pfcrt* polymorphisms. The IC_50_s of wt (wild type) and mut (mutant type) was significantly different (*P* < 0.001). The horizontal lines show the mean and the upper and lower 95% confidence limits

As the *pfcrt* K76T and A220S mutations were highly prevalent, with only a single mutant haplotype present at codons 72–76, isolates were separated into two groups, wild type (wt) and mutant type (mut) prior to in vitro drug testing (Fig. 2). There were significant differences in mean IC_50_s between the two groups (*Z* = -4.27, *P* < 0.001), the value of mut group being significantly higher than the wt group. In the single isolate which displayed reduced sensitivity to CQ after culture adaptation, there appeared to be a change from a mixed WT/mutant haplotype at *pfcrt* to a pure mutant haplotype following culture adaptation. Of the ten isolates with drug resistance associated mutations in *pfcrt*, two were classified as MR and eight as HR. However, the IC_50_s of isolates with wild type *pfcrt*, 17 were classified as S, four as MR and one as HR.

### Comparison of current *pfcrt* K76T prevalence with previously published data

Due to its rapid decline in efficiency, CQ was withdrawn as the first-line treatment for *P. falciparum* malaria from the majority of Africa between 1998 and 2008. The prevalence of the *pfcrt* K76T mutation in isolates typed in this study was compared with previously reported prevalence recorded after the official withdrawal of CQ from most of Africa. On the whole, the frequency of the CQ-resistant *pfcrt* 76T has decreased through time, although CQ sensitivity recovery trends show regional variability. Table 3 summarizes the changes in the prevalence of *pfcrt* 76T–carrying isolates after cessation of CQ use in five African countries, including Angola [28, 29], Congo [29–31], Equatorial Guinea [29, 32–34], Ghana [29, 35–37] and Nigeria [29, 38, 39] (countries contributing ≥20 isolates in the present study). The prevalence of *pfcrt* 76T–carrying isolates generally reduces through time, except in Equatorial Guinea, which shows large variation within a similar period [29, 32].

**Table 3:** Comparison with the prevalence of *pfcrt* K76T from previous studies

Country	Time of CQ withdrawal [2]	Mutation rate (%)	Sample size	Year	Reference	*P-*value (*χ* ^2^; *df*)
Angola	2006	97.0	102	2007	[28]	*P* < 0.001 (113.6; 3)
		63.5	430	2010	[28]	
		47.6	42	2011–2014	This study	
		25.7	101	2012–2015	[29]	
Congo	2006	97.0	83	2005	[31]	*P* < 0.001 (29.8; 3)
		92.0	51	2009–2010	[30]	
		80	20	2011–2014	This study	
		52.2	23	2012–2015	[29]	
Equatorial Guinea	2004	72.0	297	2005	[33]	*P* < 0.001 (274.0; 4)
		30	244	2013	[34]	
		98.7	151	2013–2014	[32]	
		31.9	47	2011–2014	This study	
		14.7	75	2012–2015	[29]	
Ghana	2004	59.3	27	2004–2006	[37]	*P* = 0.001 (19.8; 4)
		47.8	178	2007–2008	[35]	
		48.3	60	2010	[36]	
		15.0	20	2011–2014	This study	
		13.0	23	2012–2015	[29]	
Nigeria	2005	88.0	116	2004–2005	[38]	*P* < 0.001 (99.0; 3)
		95.8	119	2007–2008	[39]	
		46.9	32	2011–2014	This study	
		41.9	74	2012–2015	[29]	

*Note*: Partial studies were selected for comparison, and mixed infections were considered as mutant. Mutation rates among different years were compared, and the results indicate significant changes over time (*P* < 0.05)

## Discussion

In the present study, ex vivo and in vitro CQ sensitivity assays were performed with fresh and culture-adapted isolates of *P. falciparum*. Although continuous in vitro culture can result in the selection of a subpopulation of parasites, nine of the ten samples assayed by both ex vivo and in vitro tests showed consistent results between them. Therefore, using culture-adapted isolates in in vitro assays, although occasionally misleading in some cases, may be an effective way of monitoring CQ susceptibility, especially in areas with low prevalence of CQ resistance. Chloroquine susceptibility of the 32 isolates assayed could be categorized into three levels, including S (17 isolates), MR (6) and HR (9), but could also be separated into sensitive and resistant categories with the cut-off value being an IC_50_ of 100 nM. Using this latter criterion, eight of ten isolates carrying the *pfcrt* K76T mutation was defined as resistant, along with one isolate that was wild type at *pfcrt* but which displayed a CQ IC_50_ of 124.8 nM. Additionally, IC_50_ values did not vary significantly between years of isolation.

To date, mutations in the *pfcrt* gene are the most reliable molecular markers for CQR. We compared the in vitro CQ susceptibility of 32 isolates with point mutations in the *pfcrt* gene. Two main haplotypes were found at *pfcrt* codons 72–76; CVMNK (wt) and CVIET (mut), with the latter haplotype strongly associated with the A220S *pfcrt* mutation, consistent with previous reports [40, 41]. The IC_50_s of the majority of isolates carrying the wt haplotype were lower than 50 nM. In contrast, the isolates carrying mutations were higher than 50 nM, and 80% (8/10) were above 100 nM. Interestingly, one fresh clinical isolate was typed as a mixed genotype and was deemed sensitive by ex vivo testing, but switched to a pure mut haplotype and was shown to be CQ resistant following culture adaptation. In addition, one isolate that was wild type at the *pfcrt* locus had a CQ IC_50_ of 124.8 nM, which may indicate the influence of factors other than *pfcrt* haplotype in CQ resistance, and so warrants further study. In summary, our results indicate a strong association between *pfcrt* genotype and drug sensitivity, and are consistent with previous studies [42].

Due to the widespread occurrence of drug resistance, most African countries withdrew CQ as a first line drug for the treatment of *P. falciparum* malaria between 1998 and 2008 [2]. Following the withdrawal of CQ, complete or partial reversion to CQS alleles have been reported in some countries, such as Malawi [9, 43] and Tanzania [44]. Consistent with this, all of the isolates from these two countries considered in this work were found to be wild-type at the *pfcrt* locus. However, in contrast to a recent report from Zambia that found no chloroquine-resistant genotypes in 302 isolates [45], we found one resistant mutant in a total of eight isolates from this country. In the Democratic Republic of the Congo and the Republic of the Congo, two neighboring countries in Central Africa, CQ was withdrawn in 2001 and 2006, respectively. We found that the prevalence of the *pfcrt* mut haplotype was 18.2 and 60% in these two countries, respectively, showing a good correlation between length of time since cessation of CQ use, and the prevalence of *pfcrt* wt strains.

In the present study, analysis of the *pfcrt* gene indicated that CQ sensitive parasites were more common in the East, South and North Africa than in the West and Central Africa. Chloroquine resistance was first identified in East Africa in the late 1970s [3, 46], and countries in this region were the first to change their first line treatments from CQ to other antimalarial drugs. South Africa was the first country to recommend artemisinin-based combination therapies (ACTs). Our results confirm previous observations that a reduction in drug pressure is linked to the return of CQ sensitivity [8]. The prevalence of resistant parasites can vary greatly between closely situated countries, such as Ghana and Liberia in West Africa. Even though CQ was withdrawn from these two countries in 2006, the prevalence of *pfcrt* mut haplotypes were quite different (10.0 and 92.9%, respectively). Although ACTs were introduced at the same time in these two countries, the partner drug of artemisinin in combination therapies plays an important role in *pfcrt* mutant selection. In addition, the prevalence of *pfcrt* 76T isolates showed large variation within Equatorial Guinea between three studies carried out at similar times, of which ours is the latest. This suggests that while CQ resistance may be expected to drop following the removal of selective pressure, other factors can affect parasite population dynamics, and the prevalence of drug resistance parasites can vary greatly between locations [47].

## Conclusions

This study confirms that there is strong association between *pfcrt* haplotype and drug sensitivity, and that reduction in drug pressure may lead to an increase in CQ susceptibility. The prevalence of the *pfcrt* wild-type haplotype in the East, South and North Africa were higher than in the West and Central areas, but varied greatly between countries. While CQ resistance may be expected to drop after the removal of selective pressure, others factors probably affect this process, casting doubt on whether resistant parasites would ever disappear altogether in the absence of drug pressure.

## Abbreviations

CI: Confidence interval
CQ: Chloroquine
CQR: Chloroquine resistance
HR: High resistance
IC_50_: 50% inhibitory concentration
MR: Medium resistance
mut: Mutant type
*pfcrt*: *Plasmodium falciparum* chloroquine resistance transporter gene
S: Sensitive
SP: Sulfadoxine-pyrimethmine
wt: Wild type
